# Abnormal Notochord Branching Is Associated with Foregut Malformations in the Adriamycin Treated Mouse Model

**DOI:** 10.1371/journal.pone.0027635

**Published:** 2011-11-21

**Authors:** Piotr Hajduk, Hideaki Sato, Prem Puri, Paula Murphy

**Affiliations:** 1 Zoology Department, School of Natural Sciences, University of Dublin, Trinity College, Dublin, Ireland; 2 National Children's Research Centre, Our Lady's Children's Hospital, Crumlin, Dublin, Ireland; Instituto de Medicina Molecular, Portugal

## Abstract

Oesophageal atresia (OA) and tracheooesophageal fistula (TOF) are relatively common human congenital malformations of the foregut where the oesophagus does not connect with the stomach and there is an abnormal connection between the stomach and the respiratory tract. They require immediate corrective surgery and have an impact on the future health of the individual. These abnormalities are mimicked by exposure of rat and mouse embryos in utero to the drug adriamycin. The causes of OA/TOF during human development are not known, however a number of mouse mutants where different signalling pathways are directly affected, show similar abnormalities, implicating multiple and complex signalling mechanisms. The similarities in developmental outcome seen in human infants and in the adriamycin treated mouse model underline the potential of this model to unravel the early embryological events and further our understanding of the processes disturbed, leading to such abnormalities. Here we report a systematic study of the foregut and adjacent tissues in embryos treated with adriamycin at E7 and E8 and analysed between E9 and E12, comparing morphology in 3D in 149 specimens. We describe a spectrum of 8 defects, the most common of which is ventral displacement and branching of the notochord (in 94% of embryos at E10) and a close spatial correspondence between the site of notochord branching and defects of the foregut. In addition gene expression analysis shows altered dorso-ventral foregut patterning in the vicinity of notochord branches. This study shows a number of features of the adriamycin mouse model not previously reported, implicates the notochord as a primary site of disturbance in such abnormalities and underlines the importance of the model to further address the mechanistic basis of foregut congenital abnormalities.

## Introduction

Congenital malformations of the foregut are common in humans and represent a challenge to the paediatric surgeon both in terms of surgical repair and the management of long term morbidity [1]. Oesophageal atresia (OA) encompasses a group of congenital anomalies where the oesophagus does not connect with the stomach. The most common form of the condition is OA with distal tracheooesophageal fistula (TOF) where affected newborn babies have an oesophagus that ends blindly and an abnormal communication, or fistula, between the trachea and the stomach [2]–[5]. It is one of the most life-threatening anomalies in a newborn baby. The incidence of OA with or without a fistula is reported to be 1 in 2500 to 1 in 4500 live births [2], [3]. Although a number of theories have been proposed to explain the occurrence of foregut malformations [6], [7], little is known about the aetiology of these defects. The high incidence of associated anomalies in OA points to a very early disturbance of the developing embryo.

The precise steps in normal development of the foregut that are disturbed in OA/TOF are unknown but some new insights are emerging from the study of animal models. The foregut develops from the embryonic endoderm that involutes early from the most anterior part of the primitive streak initially forming a sheath of cells lying ventral to the mesoderm and ectoderm germ layers [8]. The anterior endoderm folds over posteriorly to form a diverticulum called the foregut pocket that pushes under the headfold while more posterior regions of the gut primordium fold laterally, as the mouse embryo turns [9]. Initially the endoderm is in very close proximity to the notochordal plate but from about E9 the notochord delaminates from the endoderm in the mid regions of the embryo and by about E9.5 it is separated from the anterior foregut by mesenchyme, becoming more closely associated dorsally with the neural folds/neural tube [10] where it is well known to contribute to dorso-ventral (DV) patterning of the developing central nervous system and laterally to patterning of the somites. Signals from the notochord are also important in patterning the foregut and its associated mesenchyme ventrally [11].

Both the respiratory and digestive anlagen arise from the foregut; the trachea forming ventrally and the oesophagus dorsally. The first morphological evidence of this is at E9.5 with the appearance of two ventrolateral bulges which subsequently elongate to form the bronchi and lung buds in conjunction with surrounding mesenchyme [12], [13]. Anterior to this, the single foregut diverticulum septates laterally to separate the trachea and oesophagus [12]–[14]. There is some disagreement in the literature about how the process of septation progresses. Recent work measuring the respective lengths of the undivided and divided regions of the foregut overtime indicates that septation progresses anteriorly from an initial point [15]. Although similar work from another group concluded that progressive separation is achieved through posterior elongation from the point of septation [16], it is clear that separation is presaged and accompanied by differentiation of foregut cells distinguishing dorsal (e.g. Sox2 expressing) and ventral (e.g. Nkx2.1 expressing) aspects that will form digestive and respiratory tissues respectively [15], [17], [18]. Development of the lung primordia progresses with extension of the bronchial stalks and stereotypic branching and budding occurs as the bronchial and bronchiolar tubules form between approximately E11 and E16 [19].

A valuable animal model of OA and TOF was developed in the rat [20], [21] following the discovery that treatment of pregnant females with the drug Adriamycin led to similar malformations [22]. However, given that the mouse is the developmental biologist's mammal of choice, providing greater availability of molecular tools and techniques, and transferable knowledge from mutant mice, Adriamycin treated mouse models displaying OA/TOF were developed [23], [24]. Adriamycin treatment of pregnant female mice was found to produce a spectrum of tracheo-oesophageal malformations in embryos: upper pouch oesophageal atresia, complete laryngotracheo-oesophageal cleft [23], [24], and tracheal agenesis [24]. All of these malformations occurred with TOF [24], abnormal notochord branching [25] and small globular stomach [23]. The models have been used to facilitate research toward understanding the developmental pathogenesis of OA/TOF [15], [23]–[29].

A number of mouse mutants replicate aspects of OA/TOF shown in the adriamycin model indicating that complex signalling events are involved in normal foregut development and disturbed in OA/TOF. Sonic hedgehog (Shh) signalling is implicated since Shh null mutants display OA/TOF with an increase in apoptosis in the lung mesenchyme [30]. Inactivation of the Noggin gene (Nog^−/−^) implicates BMP signalling since Nog null embryos replicate notochord branching defects and OA/TOF with associated effects on lung branching morphogenesis [27], [31]. The morphological similarity of foregut abnormalities seen on the one hand in a number of mutants with different genetic lesions and on the other produced by adriamycin treatment, indicate that common processes are being disturbed. The adrimaycin mouse model, therefore, may allow us to understand the mechanisms disturbed during the pathogenesis of OA/TOF.

While analysis of abnormalities in the adriamycin treated mouse model has provided some insight into the pathogenesis of OA/TOF, studies to date have been limited to partial viewing of the overall effects largely through histological sections. There is also variability in the effects that could not be fully captured. In the present study we use 3 dimensional (3D) imaging of whole embryos in a systematic analysis of 78 adriamycin treated and 71 control embryos across embryonic days 10 to 12 for morphological defects. Optical Projection Tomography (OPT) is a rapid technique for 3D imaging of whole biological tissue specimens recording morphology while also allowing visualization of the tissue distribution of RNA, protein or histological stains in developing organs [32]–[35]. We use OPT together with an endoderm marker (HNF3β) to visualize foregut and lung bud morphology at critical periods of development in control and adriamycin treated embryos. We also reveal defects at a molecular level using selected markers.

In addition to revealing delayed development in treated litters, this work shows the full spectrum of abnormalities and their frequency of occurrence. The abnormalities induced correlate with the spectrum of clinical presentations in newborns with OA/TOF. In addition this work reveals a close association between abnormal notochord branching seen in the mouse model and certain foregut malformations indicating a causative link between abnormalities in these neighbouring tissues. We propose that disturbances to notochord delamination and resulting abnormal notochordal signalling may be the mechanistic basis of certain foregut abnormalities.

## Materials and Methods

### Animals

Male and female CBA/Ca mice (Harlan UK, Bicester, England) were time-mated over a 4-hour period starting at 8 a.m. Identification of a vaginal plug at the end of the mating period was taken to be the start of gestation. Pregnant mice received two intraperitoneal injections on embryonic days E 7 and E8, each with 6 mg/kg Adriamycin (Doxorubicin, EBEWE Pharma Ges.m.b.H Nfg.KG, A-4866 Unterach, Austria) in 0.9% sodium chloride. Control mice received an equivalent volume of 0.9% sodium chloride. Dams were humanely killed by swift cervical dislocation between E10 and E12. The embryos were washed in phosphate buffered saline (PBS) and then fixed in 4% paraformaldehyde in RNase free PBS overnight. The embryos to be used for in situ hybridisation were stored following dehydration through a series of methanol/PBT (PBS+0.1% TritonX100) washes, in 100% methanol at −20°C. Embryos from E12 were dissected to include only the trunk region from the first branchial arch to the base of the liver to improve penetration of RNA probes and antibodies.

All experiments were carried out in compliance with current European Union regulations for animal investigation (ED86/609/EC), with prior ethical approval under license no. B100/4106 from the Department of Health, Ireland.

### Whole mount in situ hybridization and immunolocalization

Plasmids carrying cDNA clones representing Shh and Foxf1 genes were received from L. Lundh; Nkx2.1 from L. Magno and Sox2 from R. Lovell-Badge. Details of the probes used are summarised in Table 1. Antisense and sense digoxigenin (dig)-labelled RNA probes were generated from 1 µg linearised plasmid using T7, T3 or SP6 promoter sites according to insert orientation (all reaction components from Roche, Germany). RNA production and integrity was checked by running 1 µl on a 1% agarose gel. DNA template was degraded by incubation with 1 µl RNase free DNase (Roche, Germany). The *in vitro* transcribed RNA was purified on G25 columns (Amersham bioscinces, USA) as per manufacturer's instructions and stored at −20°C in an equal volume of hybridization mix. Hybridisation buffer contained 2% blocking reagent (Roche, Germany), 50% formamide, 5× saline-sodium citrate buffer (SCC), 0.5% 3-[(3-Cholamidopropyl) dimethylammonio] -2-hydroxy-1-propanesulfonate (CHAPS), 500 ug/ml Heparin, 1 ug/ml Yeast RNA, 0.1% Tween 20 and 5 mM ethylenediamine tetraacetic acid (EDTA).

**Table 1:** Details of cDNA clones used as in situ probes.

Gene	Extent of Probe on Genbank Sequence
Shh	Nucleotide 452 to 1097 on NM_009170.3
Foxf1	Nucleotide 342 to 742 on NM_010426.1
Nkx2.1	Nucleotide 65 to 563 on NM_009385.3
Sox2	Nucleotide 31 to 778 on NM_011443.3

Whole embryos stored in 100% methanol were subsequently rehydrated through a series of methanol/PBT washes. Embryos were digested with 10 µg/ml proteinase K (Roche, Germany) in PBT for 6 to 30 minutes depending on stage. Embryos were refixed in 0.2% glutaraldehyde/4%paraformaldehyde for 20 minutes. Embryos were prehybridized with hybridization buffer at 65°C overnight. The buffer was replaced with fresh hybridization buffer containing 1 µg/ml digoxigenin-labeled riboprobes, and hybridization was performed at 65°C overnight. Post-hybridization washes at 65°C were 1× 5 min in 50% formamide/5× SSC/0.5% CHAPS, then three serial 30 min washes in decreasing concentrations of formamide, SSC and CHAPS, culminating in 2× 30 min washes in 2× SSC/0.1% CHAPS and 2× 30 min in 0.2× SSC/0.1% CHAPS and 2× 10 min in TNT (100 mM Tris, pH 7.5, 150 mM NaCl, 0.1% Triton X-100) at RT. After washing, the embryos were blocked in 10% goat serum in TNT overnight and were incubated with 1/1000 dilution of antidigoxygenin antibody (Roche #11093274910, Germany) in the same blocking solution. For double labeling, embryos were simultaneously incubated with HNF3β primary antibody (07-633 Upstate Cell Signaling Solution, Lake Placid, NY) diluted 1/500 in the same blocking solution (10% goat serum (Sigma-Aldrich, Germany)) for at least 4 days at 4°C with rocking. The embryos were washed with TNT overnight and incubated with Cy3 conjugated Goat anti-rabbit IgG (111-165-144 Jackson ImmunoResearch) at 1/200 dilution overnight and washed four times for 30 mins at room temperature in TNT with rocking to remove any unbound secondary antibody. Embryos were stained colorametrically to localize RNA expression using NBT/BCIP solution (Roche, Germany).

### Optical Projection Tomography

Stained embryos were embedded in 1% low melting point agarose (Sigma-Aldrich, Germany) in water, attached to a metal mount, dehydrated in 100% methanol overnight and cleared in benzyl benzoate/benzyl alcohol (1∶2) for at least 5 hours [32], [33]. Projected images of the specimens were captured in a prototype OPT scanner constructed at the MRC Human Genetics Unit, Edinburgh and installed in the Zoology Department Trinity College Dublin. A Q imaging Retiga Exi camera was used to record images through a 360° rotation of the specimen viewed through a Leica MZ FLIII microscope with a plan 0.5× objective. At least two scans were performed for each specimen, 1) using UV light and a TXR filter (560/40 nm excitation, 610LP nm emission) and, 2) for colorimetric staining using visible light and a 700 nm filter for very intense staining. The raw data (400 projected images) from each of the scans were loaded onto a Linux workstation, reconstructed using a set of programmes provided by the Edinburgh Mouse Atlas Project (EMAP) and analysed using custom made software MA3DView and MAPaint.

### Analysis of 3D digital data

Surface rendering of the 3D data was carried out using a suite of Visualization Toolkit (VTK) scripts and software. This recreated the surface of the embryos, so that external morphological characteristics such as the shape of the branchial arches or limbs could be used to attribute each embryo to a Theiler Stage [36] to compare development rate in control and treated embryos and for more accurate comparisons.

3D reconstructions of control and treated embryos following HNF3β immunolocalisation and OPT scaning were analysed either by viewing 3D representations of HNF3β stained tissue domains (using visualisation tool kit (vtk) software), or by virtually cutting sections through the 3D reconstructions to view the internal morphology of the developing tissues. Virtual sections through the 3D reconstructions in the appropriate planes which captured the morphological characteristics of the embryos and gene expression were selected and scored and/or measured. Sections from the two different channels (Texas Red and Visible) were then merged with IPLab software, in order to see the relationship between the expression patterns and the morphology.

### Statistics

Statistical analysis of quantitative data was performed using the Mantel-Haenszel X-squared test as appropriate using Rv2.10.0.

## Results

### Delayed development in adriamycin treated embryos

**Figure 1:**
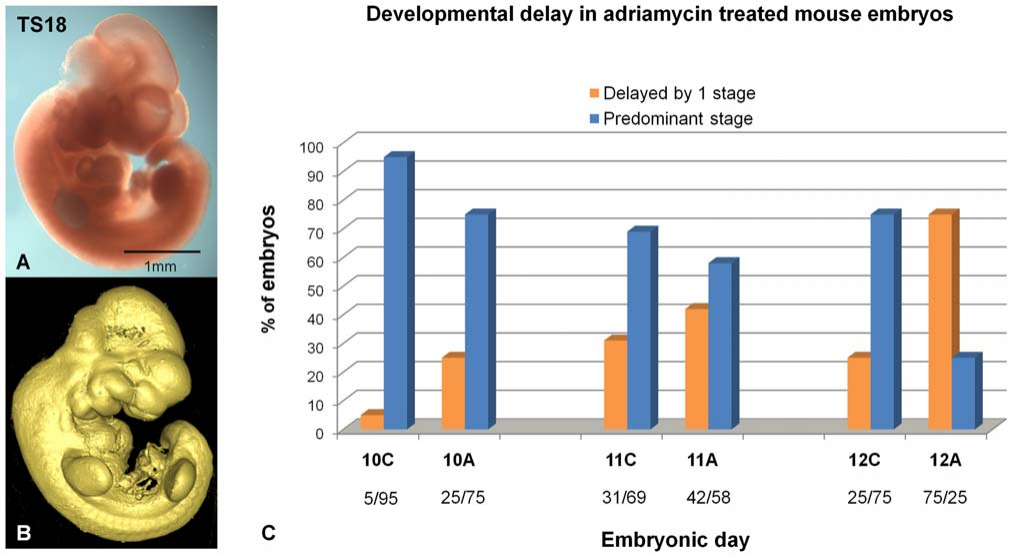
Theiler staging of adriamycin treated and control embryos revealed a significant delay in development following adriamycin treatment. (A) External view of an E11 control embryo. (B) Surface rendered view of the OPT 3D reconstruction of the same embryo showing morphological features used for staging purposes, staged as TS18. Scale bar as indicated. (C) Graphical representation of staging analysis across days E10 to E12 (n = 44; n = 72; n = 26 respectively) the percentage of embryos collected on each day of development that fall into the predominant stage (E10, TS16,; E11, TS18; E12, TS20) is represented in blue bars; organge bars represent the percentage of embryos delayed by 1 stage. Mantel-Haenszel X-squared tests show significant differences in the proportions of delayed embryos in treated groups versus control groups (pvalue<0.01) on each day E10, E11 and E12.

A total of 149 embryos divided by embryonic day (E10–E12) of collection and adriamycin treated or control groups were analyzed in detail from 3D digital recordings which provide a permanent record of the morphological features in 3D that could be reanalyzed and compared from multiple perspectives. Figure 1B shows the external morphological features that can be viewed from a surface representation of a typical digital recording. Each embryo was staged using Theiler criteria [36] from Theiler stage (TS) 14 to TS20. The appearance and shape of branchial arches, forebrain vesicles and anterior and posterior footplates were key morphological criteria used here. Examining the range of stages at each embryonic day for control embryos, it was found that the predominant stage at E10 was TS16, at E11 was TS18 and at E12 was TS20. At E10, 5% of control embryos were staged below TS16 (TS15) compared to 25% adriamycin treated embryos. At E11, 31% of control embryos were staged below TS18 (TS17) in comparison to 42% in the adriamycin treated group. At E12, 25% of control embryos were staged below TS20 (TS19) compared to 75% in the adriamycin treated group. Statistical analysis using the Mantel-Haenszel X-squared test showed a significant delay (p value<0.01) in development of the treated embryos in comparison to controls across all investigated stages (Figure 1).

### A spectrum of foregut abnormalities are caused by adriamycin treatment

**Figure 2:**
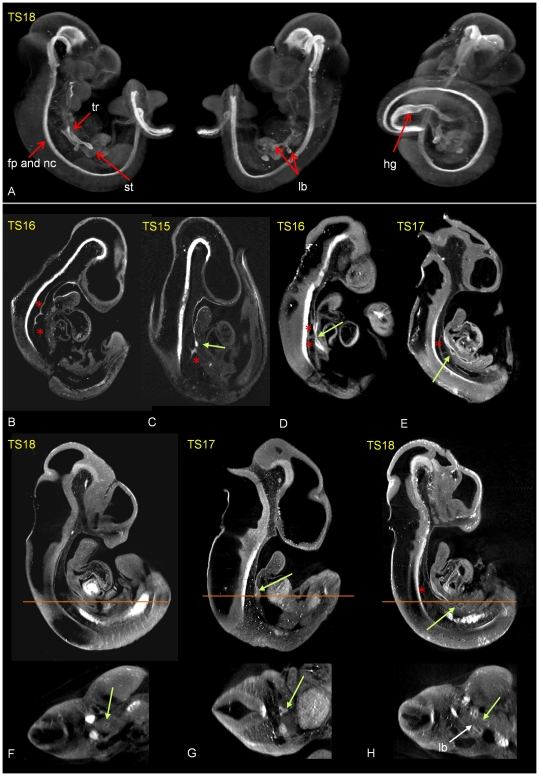
Morphological analysis of the spectrum of effects of adriamycin treatment on foregut and notochord development. (A) External views from different angles of a 3D reconstruction of a control embryo showing HNF3β labelled endoderm, notochord and floor plate (in white). (B–H) Virtual sections through 3D reconstructions of adriamycin treated embryos immunostained for HNF3β showing examples of the morphological abnormalities observed. Abnormal notochord branching (red stars) and foregut abnormalities (green arrows) are indicated. (1) abnormal notochord delamination, ventral displacement and branching (B, C, D, E; red stars), (2) foregut atresia (C), (3) foregut stenosis (D), (4) OA with upper pouch (E), (5) LTEC (F), (6) tracheal atresia (G), (8) fistula from the right bronchii (H). Absence of stomach (7) can't be shown. The images underneath are transverse sections in the planes indicated. lb; lung bud, fp; floor plate, nc; notochord, hg; hindgut, st; stomach.

To assess the spectrum of effects on foregut development in adriamycin treated embryos 78 adrimaycin treated embryos and 71 control embryos from E10 to E12 were immunostained to localize the endoderm and notochord marker HNF3β and OPT scanned. HNF3β is a forkhead domain transcription factor specifically expressed in floor plate, notochord and endoderm tissue including the foregut and lung buds, frequently used as an endoderm marker [32]. Figure 2A shows an example of HNF3β labelled tissues in a control embryo, viewed following whole mount detection and OPT scanning. The 3D digital recordings of each embryo were analyzed to assess the morphology of the foregut.

Eight morphological alterations were noted in adriamycin treated embryos: (1) abnormal branching of the notochord, (2) atresia of the foregut (complete discontinuity), (3) foregut stenosis (narrowing of the lumen), (4) oesophageal atresia (OA) with upper pouch, showing a blind dorsal pouch of the anterior foregut, (5) total laryngotracheo-oesophageal cleft (LTEC), characterized by the absence of a septum dividing the trachea and oesophagus, (6) tracheal atresia, characterized by a blind ventral pouch of anterior foregut, (7) stomach agenesis, (8) fistula from the carina or bronchi, where there is a narrow connection between the respiratory tract and the stomach. Table 2 summarises the percentage incidence of each of the abnormalities above, across the days of collection. Figure 2 shows examples of each abnormality.

**Table 2:** The spectrum of adriamycin induced abnormalities observed showing % incidence at each embryonic day.

Adriamycin induced abnormalities	E10C (24)	E10A (23)	E11C (35)	E11A (40)	E12C (12)	E12A (15)
**1.** Notochord branching	0/24 0%	18/19 94%	0/35 0%	33/38 87%	0/12 0%	9/14 64%
**2.** Foregut atresia	0/24 0%	1/15 7%	0/35 0%	2/35 6%	0/12 0%	0/11 0%
**3.** Foregut stenosis	0/24 0%	5/15 33%	0/35 0%	3/35 9%	0/12 0%	1/10 10%
**4.** OA with upper pouch	0/24 0%	2/15 13%	0/35 0%	5/35 14%	0/12 0%	6/10 60%
**5.** LTEC	0/24 0%	4/15 27%	0/35 0%	16/35 45%	0/12 0%	2/11 18%
**6.** Tracheal atresia	0/24 0%	1/15 7%	0/35 0%	8/34 24%	0/12 0%	0/11 0%
**7.** Stomach agenesis	0/24 0%	1/13 8%	0/35 0%	3/33 9%	0/12 0%	2/11 18%
**8.** Fistula from carina or bronchii	0/24 0%	1/15 7%	0/35 0%	22/35 63%	0/12 0%	6/11 55%

The number of embryos analysed at each stage for control (C) and adriamycin treated (A) conditions is given in brackets (e.g. E10C (24)). The number of embryos showing the abnormality, per scorable* embryos for that abnormality, is tabulated (*specimens were not scorable for a particular trait if the reconstuction was not clear in the relevant region).

In control groups, no anomalies were found. Most adriamycin treated embryos showed some abnormalities with only 2 embryos across stages appearing normal in all respects scored. Abnormal position (ventral displacement) and notochord branching was seen in almost all treated embryos; in 18/19 at E10. At E10 in adriamycin treated embryos, the full spectrum of foregut malformations was recorded. Foregut stenosis and LTEC were the most frequently recorded abnormalities, found in 33% and 27% respectively. Fistula was found only in 7% of the embryos. At E11 the incidence of LTEC (45%), tracheal atresia (24%) and fistula (63%), become more frequent with a striking increase in the incidence of fistula. Foregut stenosis was recorded only in 9% of cases. At E12 we noted an increased incidence of OA with posterior pouch (60%) and stomach agenesis (18%). The incidence of lower fistula was still high (55%). Foregut atresia, LTEC and tracheal atresia were recorded less frequently. The reduced incidence of the very severe anomaly of complete foregut atresia may be related to an increase in embryo reabsorption noted at this stage (not shown). The decrease in LTEC may indicate that separation of the oesophagus and trachea occurs later in some adriamycin treated embryos.

### Abnormal notochord branching in adriamycin treated embryos is associated with the location and severity of foregut abnormalities

**Figure 3:**
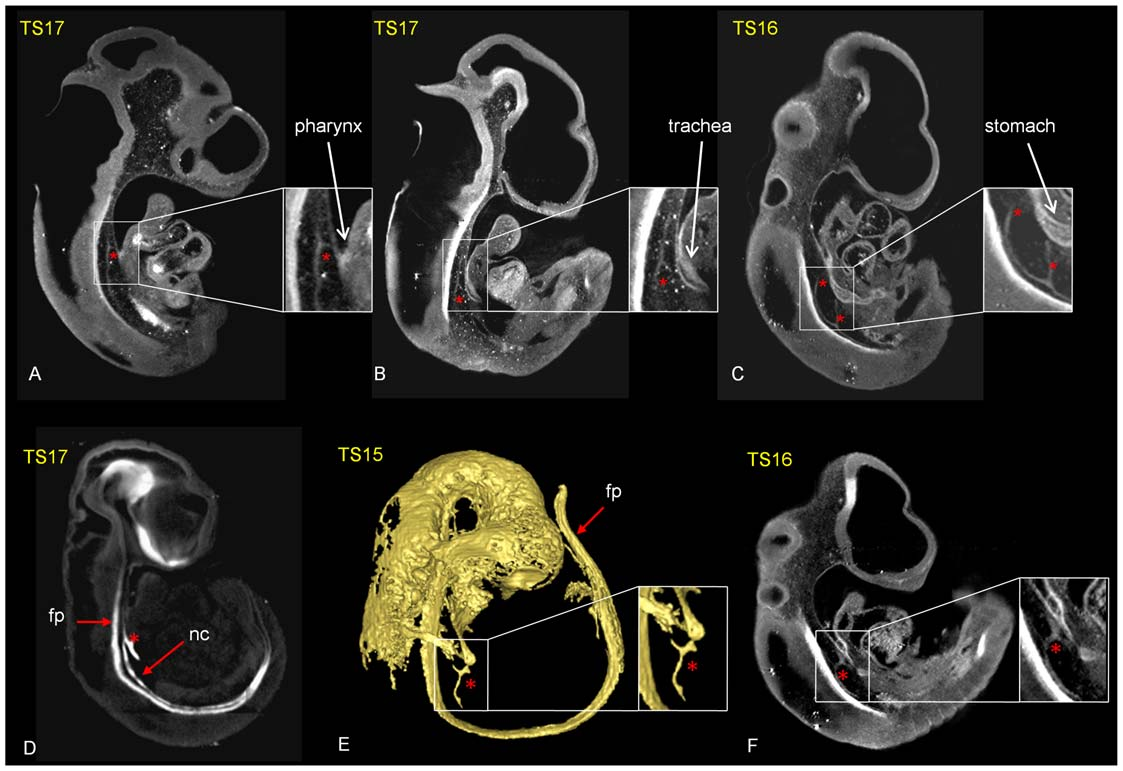
Association between notochord branching (red stars) and foregut abnormalities in 6 Adriamycin treated embryos (A–F); A–C and E,F show immunolocalisation of HNF3β, D shows Shh gene expression following whole mount in situ hybridisation. Virtual saggital sections of OPT reconstructions (A–C) demonstrate the different A-P levels of the abnormal branching notochord noted in the text; anterior to pharynx (A), at the level of the trachea (B), between the site of tracheal separation and the stomach (C). (D) shows Shh expression in an abnormally delaminated and branched notochord (red star). (E) shows a surface rendered reconstruction of HNF3β immunolocalisation illustrating association between an abnormal, heavy branch of the notochord (red star) and foregut atresia. (F) illustrates association between an abnormal, heavily branched notochord in a posterior location (red star) attached to the dorsal wall of the foregut and foregut stenosis. fp; floor plate, nc; notochord.

Abnormal notochord branching was the most frequently recorded anomaly across the stages and was most prominent at E10 (94%) and E11 (87%) respectively. In normal development, by E12 the notochord is no longer visible perhaps explaining the lower incidence of the abnormality recorded in older embryos. In addition to abnormal branches extending ventrally, the entire structure was frequently ventrally displaced with respect to the floorplate of the neural tube (Figure 2B). Notochord branching in adriamycin treated embryos was scored according to number, heaviness and positioning of the branches at E10 and E11, as well as association with foregut malformations (Table S1). The Anterior-posterior (AP) level of the branching and the number of branches could be accurately scored from virtual sections cut through multiple planes in computer reconstructed specimens. Figure 3 shows examples of sections through the reconstructions showing the position and extent of the branching. The HNF3beta positive abnormal branches seen to extend ventrally from the notochord were also shown to express the gene encoding Sonic hedgehog (Shh), an important signaling molecule produced by the notochord (Figure 3D) [37]. Notochord branching was subdivided into three categories according to AP position of occurrence: (1) anterior, at the level of the pharynx (Figure 3A), (2) more posterior, at the level of the site of tracheal separation (Figure 3B) and (3) posterior, between the site of tracheal separation and the stomach (Figure 3C). Each embryo was also scored for the appearance of foregut abnormalities to reveal any association between notochord branching and specific foregut abnormalities (Table S1). Posterior localized (level 3) heavy branching of the notochord, attached to the dorsal wall of the foregut was found more often associated with foregut atresia (2) and foregut stenosis (3) (Figure 3 D, E). More posterior branching of the notochord (level 2+3) was found to be associated with OA with upper pouch (4) and stomach agenesis (7). LTEC (5), tracheal atresia (6) and fistula (8) were associated with notochord branching at all levels. Fistula (8) is an interesting case since the abnormality is necessarily localized posteriorly, at the level of the stomach, but its occurrence is associated with branching at all levels (Table S1).

### Patterning of the foregut is disturbed at the site of abnormal notochord branches

**Figure 4:**
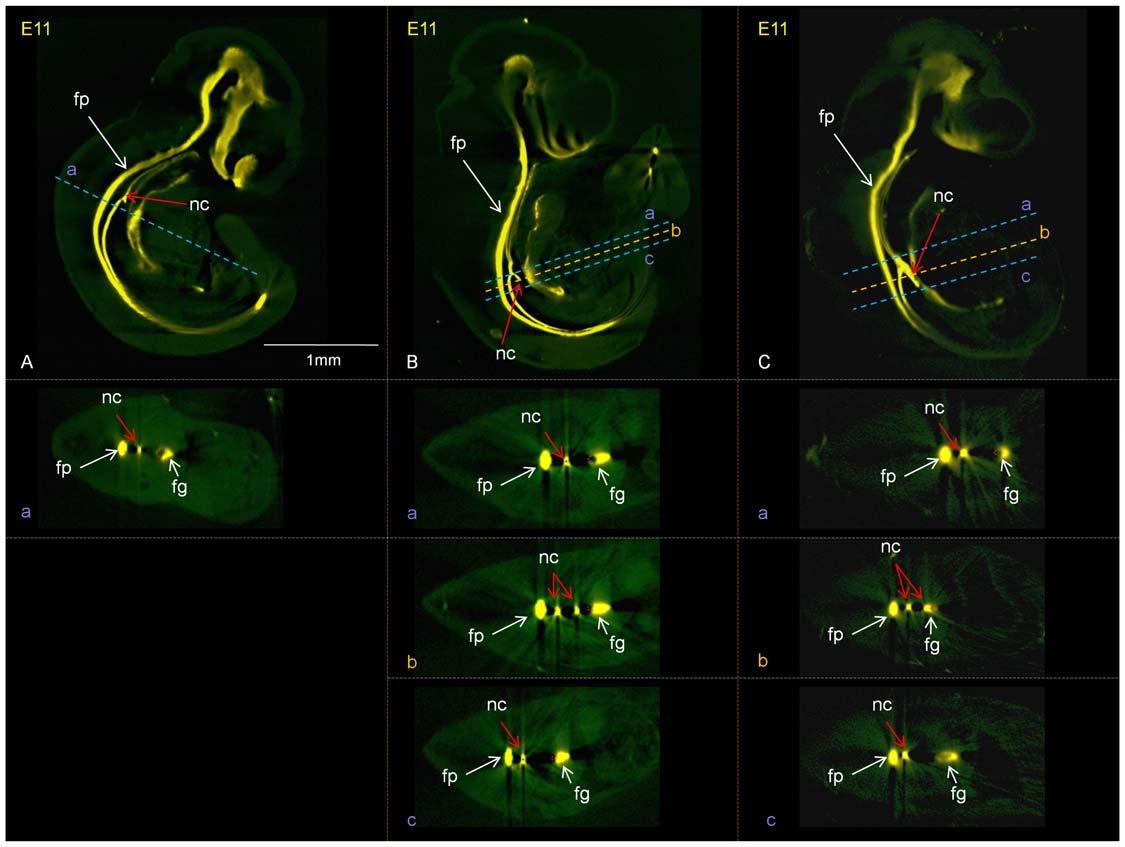
Shh expression in the branched notochord and disturbed Shh expression in the foregut following adriamycin treatment. Embryos at E11 showing notochord branches at different anterioposterior levels, different heaviness and different distances from the foregut: A) short anterior branch; the cross section in a) shows normal localization of Shh expression in the ventral foregut (dorsal foregut outlined in red). B) long narrow notochord branch. Cross section at b shows the branched notochord; cross sections at a, b and c show normal ventral restriction of Shh expression C) Heavy notochord branch adjacent to the foregut; a,b,c cross sections show abnormal notochord branch in b and abnormal expression of Shh through the dorsal foregut in c.

Shh expression in the foregut normally shifts from being expressed in the ventral endoderm of the prospective trachea prior to separation at E10.5 to being dorsally restricted to the separated oesophagus at E11.5 [26]. Adriamycin treated embryos showed a lack of dorso-ventral restriction of Shh expression at the site of an unseparated foregut at E11.5, indicating a disturbance of normal D/V patterning. We investigated if Shh expression in the foregut is specifically disturbed in the vicinity of ectopic notochord branches (Figure 4). At E11 ectopic Shh expression was seen through abnormal notochord branches of various length and thickness (Figure 4A, B, C). In addition, there was an abnormal dorsal shift of the expression in the foregut specifically at the point where a heavy ventral notochord branch was positioned adjacent to the foregut (Figure 4Cb, 4Cc). Where there was limited ventral extension of the branch (Figure 4A) or the branch was light (Figure 4B), Shh was normally restricted to the ventral endoderm as expected at this stage.

**Figure 5:**
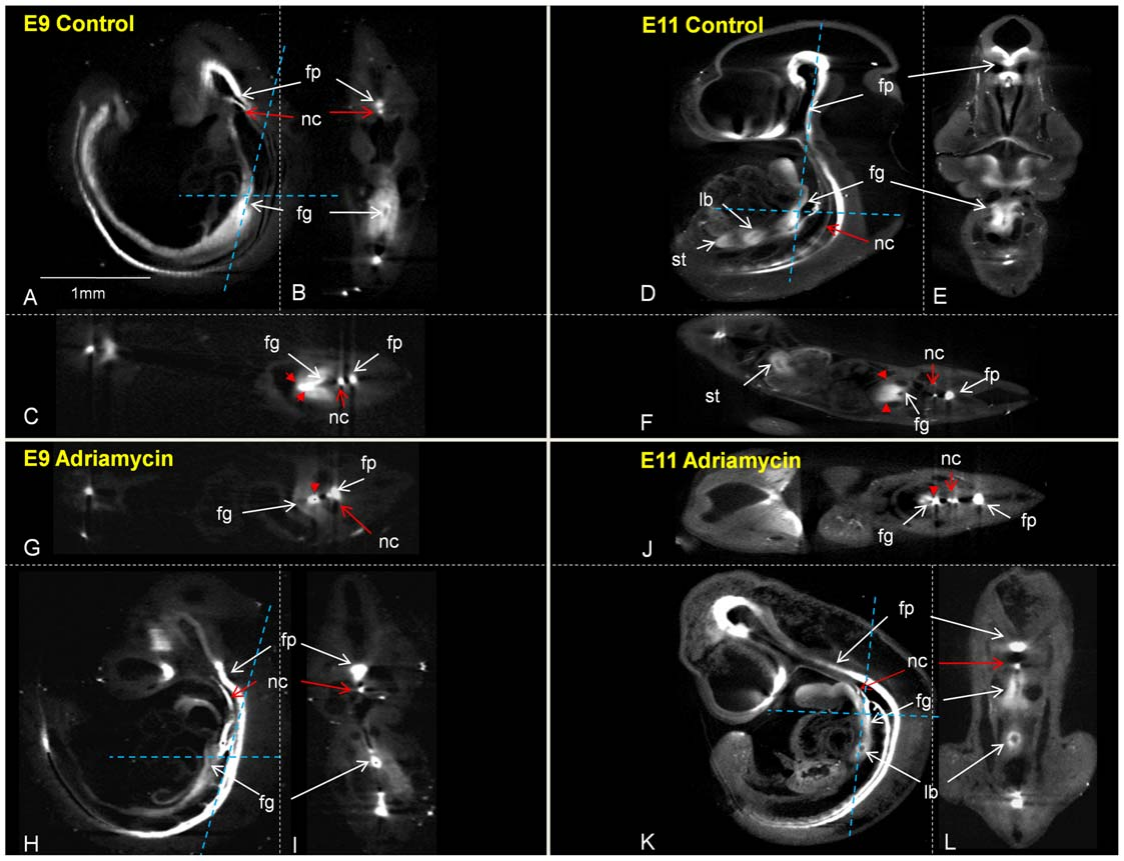
Analysis of Foxf1 expression in adrimycin treated and control embryos shows abnormal localisation in the foregut associated mesenchyme in the vicinity of ectopic notochord branches. Control (A–F) and adriamycin treated embryos (G–L) represented by virtual, sagittal (A,D,H,K), coronal (B,E,I,L) and transverse (C,F,G,J) sections of OPT reconstructions following whole mount in situ hybridisation with Foxf1 antisense probe show expression in the floor plate, notochord, foregut endoderm and associated mesenchyme. Blue dotted lines indicate the planes of coronal and transverse sections. Red arrow heads on transverse sections of control embryos at E9 and E11 (C, F) indicate the ventral bias in distribution of Foxf1 transcripts in the foregut mesenchyme. Note the absence of this pattern in treated embryos (G, J). fp; floor plate, nc; notochord, fg; foregut, lb; lung bud.

We furthermore investigated expression of the Foxf1 gene, which encodes a forkhead or winged helix trancrition factor and is expressed during organogenesis in the mesenchyme adjacent to the endodermal epithelia of the gastrointestinal tract [38], [39]. Haploinsufficiency of Foxf1 leads to a phenotype similar to Shh null mutant mice including hypoplastic lungs and lobulation defects, oesophageal and tracheal stenosis, or oesophageal atresia and tracheo-oesophageal fistula [30], [40]. We therefore examined the expression of Foxf1 in adriamycin treated and control embryos and found that mesodermal expression of the gene around the foregut of treated embryos showed less of a ventral bias and was more symmetrically distributed around the foregut, specifically in the vicinity of abnormal notochord branches (Figure 5). To further investigate the coincidence of ectopic notochord branches and abnormal dorso-ventral expression of Shh and Foxf1 in and around foregut endoderm, we measured the extent of foregut affected anterior and posterior to the notochord branch by counting the number of consecutive transverse virtual sections affected in a sample of treated embryos at E10 and E11 (Table 3). Although there was much variability in the extent of the effect, the pattern in general corroborated the example shown in Figure 4, where heavy multiple branches (** and ***) were associated with the most affected specimens (up to 490 µm) whereas single, fine branches (*) were associated with limited or no disturbance (e.g. 0, 30, 150 µm).

**Table 3:** Extent of foregut (µm) in which dorso-ventral localised expression of Shh or Foxf1 was disturbed in individual adriamycin treated embryos at E10 or E11.

Specimen	Gene expression analysed	Extent of disturbed expression pattern	Relative heaviness/proximity of notochord branch
E10 2018	Shh	260 µm	2***Y
E10 2019	Shh	100 µm	2***Y
E10 2020	Shh	230 µm	2**, 3**
E10 2054	Shh	160 µm	1*,3*
E10 1817	Shh	490 µm	3***Y
E11 1785	Shh	180 µm	1**,2**
E11 1787	Shh	30 µm	2*
E11 2025	Shh	312 µm	2***
E11 2058	Shh	112 µm	2***
E11 361	Shh	221 µm	2**
E11 362	Shh	0	1*
E10 1828	Foxf1	390 µm	2**Y
E10 2041	Foxf1	130 µm	3**
E10 2036	Foxf1	250 µm	2***Y
E10 2040	Foxf1	250 µm	3***Y
E10 2039	Foxf1	460 µm	2***,3**
E11 2046	Foxf1	53 µm	1**,2**, 3*
E11 2045	Foxf1	350 µm	3***
E11 2047	Foxf1	420 µm	1*, 2***, 2*
E11 1789	Foxf1	150 µm	2*
E11 1790	Foxf1	300 µm	2**
E11 1791	Foxf1	255 µm	2**Y

The right hand column shows the anterior/posterior position and relative heaviness and proximity of the notochord branches (as indicated in Table S1).

**Figure 6:**
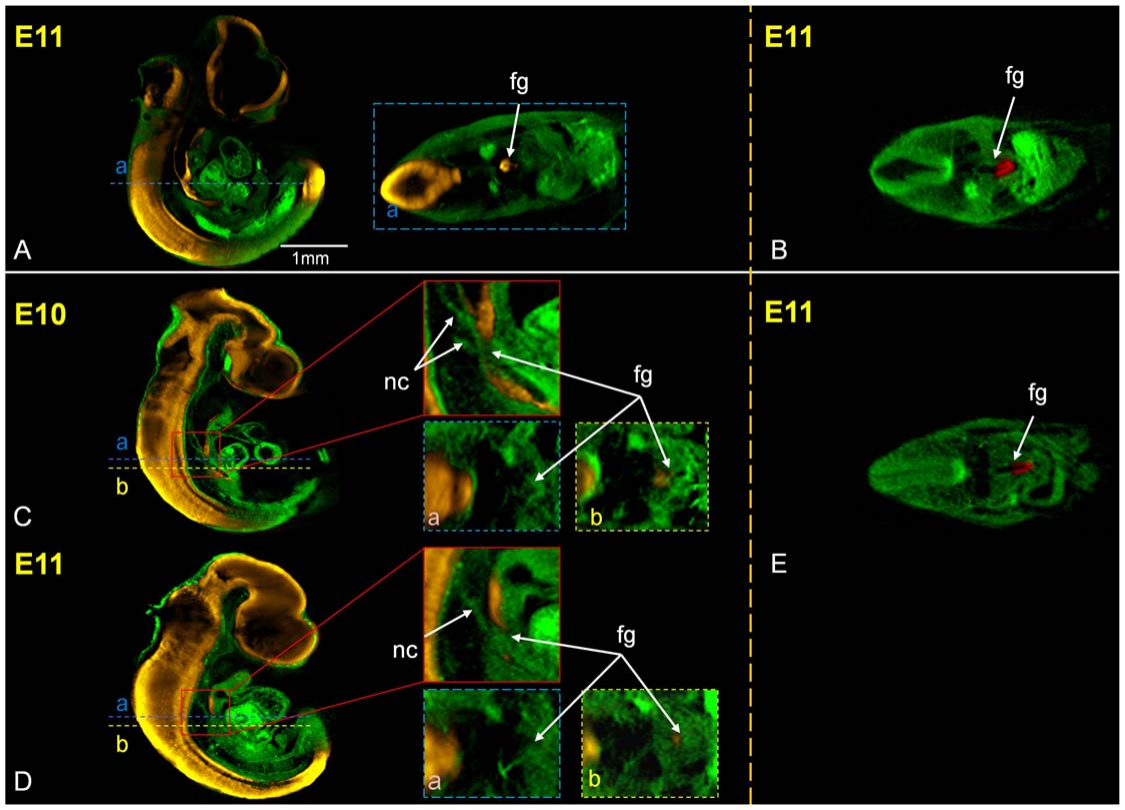
Sox2 expression in the dorsal foregut is lost at the site of abnormal notochord branches while Nkx2.1 expression remains localized to the ventral foregut. Sox2 expression (A, C and D, shown in yellow) and Nkx2.1 expression (B and E, in red) in control (A, B) and adriamycin treated (C, D and E) embryos at embryonic days 10 and 11 as indicated. Sagittal sections are shown on the left of A, C and D with transverse sections on the right in A (in plane indicated) and in B and E. Higher magnification views at a notochord branch are bounded in red in C and D. Transverse section in the planes indicated as a and b at and below the site of a notochord branch are shown bounded in blue and yellow respectively. nc; notochord, fg; foregut.

Dorsoventral patterning of the foregut is also reflected in dorsal expression of Sox2 and ventral expression of Nkx2.1 and in Nkx2.1 null embryos Sox2 expression spreads ventrally accompanied by failure of trachea-oesophageal separation [15], [17], [18]. We therefore examined Sox2 and Nkx2.1 gene expression in adriamycin and control embryos and found that while Nkx2.1 expression was normally confined to the ventral domain, dorsal Sox2 expression was absent in the vicinity of an ectopic notochord branch (Figure 6).

### Tracheal separation and lung branching in adriamycin treated embryos

**Figure 7:**
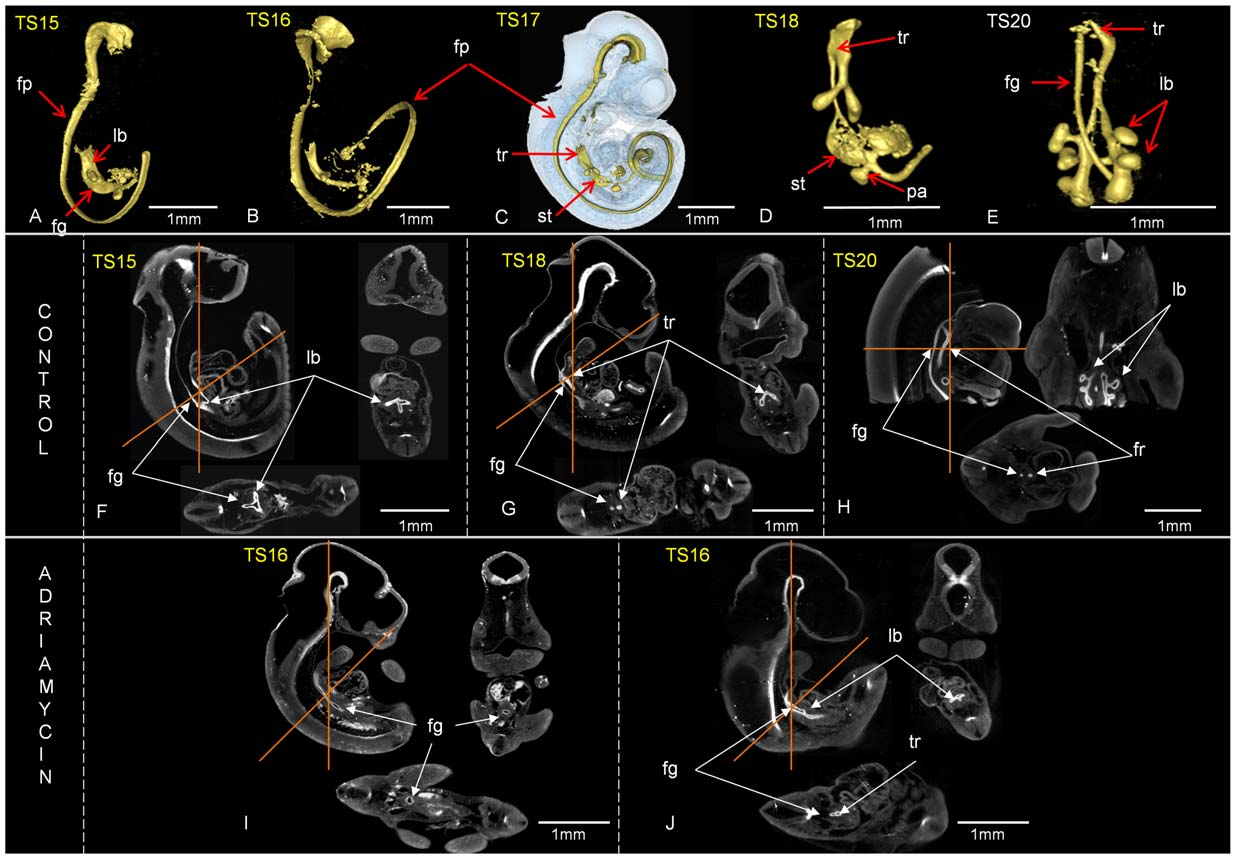
Abnormal tracheo-oesophageal separation and lung morphogenesis in adriamycin treated embryos. A–E; normal tracheo-oesophageal separation and lung bud branching morphogenesis from TS15- TS20 revealed through 3D surface representation of HNF3β immunolocalisation. C shows surface representation of the HNF3β positive domain (in yellow) in the context of the whole embryo (blue, transparent). Sections through 3D reconstructions in saggital (main images), coronal (to the right) and transverse (below) orientations of control (F–H) and adriamycin treated (I–J) embryos. The position of coronal and transverse sections are indicated with red lines. At TS15 ventrolateral bulges on the foregut indicate the first appearance of the lung buds (A and F). At TS16 and TS17 the lung buds elongate (B, C). From TS17 separation of the trachea begins with extended separation at subsequent stages (C, D, E and G, H). Branching morphogenesis of the lung buds is extensive at TS20 (E and H). Scale bars as indicated. Examples of adriamycin treated embryos (I–J), at TS16 show abnormal oval widening of the foregut instead of lung primordia (I). The site of lung bud separation from the foregut is more posterior in comparison to control embryos (I). Abnormal tracheal separation; where the trachea remains close to the ventral wall of the foregut (J). fg; foregut, lb; lung bud, tr; trachea, fp; floor plate, lb; lung bud, fg; foregut, tr; trachea.

Normal lung development could be followed in control embryos using OPT imagining and the endoderm marker HNF3β (Figure 7). The lung bud primordia start forming at TS15 (Figure 7A) with very specific triangular widening of the foregut (Figure 7F). From TS16–TS17 the oesophagus starts to separate from the trachea (Figure 7B,C,G). Further elongation of the trachea and lung bud branching morphogenesis were recorded from TS18 up to TS20 (Figure 7D,E,H). This normal progression is consistent with previous findings [19], [41]. Each adriamycin treated embryo was also staged according to external morphological criteria [36] and the extent of tracheal separation and lung bud branching was assessed through viewing virtual saggital, coronal and transverse sections. In adriamycin treated embryos, otherwise morphologically staged at TS15 and TS16, lung primordia were not present in 33% (4 out of 12) or present as only an oval widening of the foregut in 73% (8 out of 12) (Figure 7I) of specimens. In treated embryos staged as TS17 only 20% (3 out of 15) of the embryos presented with separation of the trachea from the oesophagus and lung bud division, 53% (8 out of 15) of the embryos presented without tracheal separation but with appearance of lung buds and 27% (4 out of 15) of the embryos presented with only oval widening of the foregut.

In treated embryos staged as TS18, 0 out of 22 embryos had normal tracheo-oesophageal separation, 18% (4 out of 22) of the embryos had lung bud division and 82% (18 out of 22) oval or transverse widening of the foregut. In treated embryos staged as TS19, 14% (1 out of 7) of the embryos had normal tracheo-oesophageal separation and lung bud division, 29% (2 out of 7) of the embryos presented with lung bud division and 57% (4 out of 7) of the embryos showed only triangular widening of the foregut.

In treated embryos staged as TS20, 20% (1 out of 5) of the embryos presented with separation of the trachea from the foregut and lung bud division, 60% (3 out of 5) of the embryos had lung bud division but no tracheal separation and 20% presented with only oval widening of the foregut.

From the above it is clear that only a minority of adriamycin treated specimens, even at TS20, show separation of the trachea and oesophagus. Even when separation was noted, the distance between the dorsal wall of the trachea and the ventral wall of the foregut was reduced and lung buds separated more posteriorly in comparison to control embryos (Figure 7J).

## Discussion

**Figure 8:**
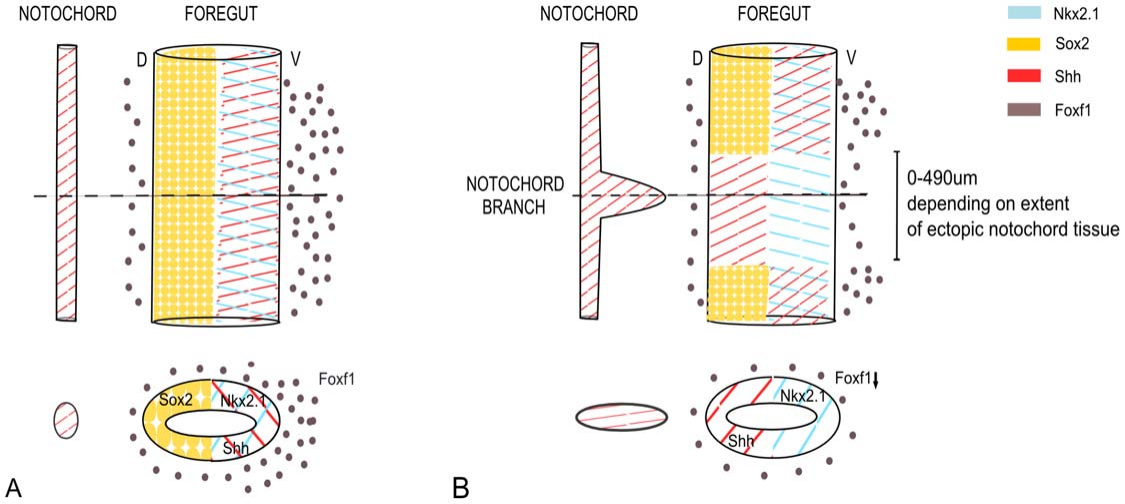
Schematic representation summarising the observed effects of adriamycin treatment on dorso-ventral patterning of the foregut, specifially in the vicinity of a ventral notochord branch. A and B summarise the observed expression of Shh, Foxf1, Sox2 and Nkx2.1 in control and treated embryos respectively. The upper drawings represent lateral views of the notochord and adjacent foregut; the lower drawings represent transverse sections in the plane indicated above. In the vicinity of a Shh expressing notochord branch, Sox2 expression is lost in the dorsal foregut, Shh expression is abnormally expressed dorsally and the normal ventral polarisation of Foxf1 expression in the adjacent mesenchyme is lost.

Here we show systematic, detailed, morphological analysis of a substantial number of mouse embryos exposed to adriamycin *in utero*, an established model of foregut congenital abnormalities termed OA/TOF. We reveal delayed development and a full spectrum of abnormalities and their frequency of occurrence in adriamycin treated embryos. A very frequent abnormality noted was ventral displacement and branching of the notochord in 94% of E10 and 87% of E11 embryos. Our whole 3D embryo imaging approach allowed an association to be revealed between the position and severity of notochord branching and particular foregut abnormalities suggesting a mechanistic link between the effects on the notochord and the foregut. This is further emphasized by the observations of altered dorso-ventral patterning of the foregut, in terms of localized Shh, Foxf1 and Sox2 gene expression, specifically in the vicinity of a notochord branch (summarized in Figure 8).

Like all embryonic events, foregut and lung development involve complex changes in the shape and structure of tissues over short periods of time. It is therefore important to view and analyze normal and altered events in three dimensions. Although previous studies have used 3D reconstructions from serial embryo sections [16], [42], [43], OPT scanning offers several unique advantages over section reconstructions and permitted a more complete analysis of a relatively large number of specimens in this study. First, because OPT scanning involves direct 3D image capture, the approach does not suffer from the difficulties of loss of tissue and distortion suffered in section reconstruction methods [32]. Second, OPT scanning is relatively rapid and significantly less time consuming that sectioning specimens, permitting a relatively large number of specimens to be analyzed. Third, because OPT allows capture of colorimetric and fluorescent stains [32], [44], molecular markers can be used to visualize specific cells and tissues, as well as gene expression patterns; this was used to advantage visualizing the endoderm and notochord marker HNF3β. In the approach used here, a permanent 3D computational representation of each treated and control embryo was recorded and could be analyzed repeatedly from multiple perspectives. For example, using MAPaint, software, developed by the Edinburgh Mouse Atlas Project (EMAP; http://genex.hgu.mrc.ac.uk), the 3D computer specimens could be digitally sectioned in any arbitrary plane of orientation and position, with multiple planes viewed simultaneously. We created 3D digital records of 149 control and adriamycin treated embryos across early stages of development from E10 to E12. The combined use of the HNF3β tissue marker and full 3D imaging, allowed a more comprehensive analysis of the full spectrum of abnormalities found in adriamycin exposed embryos.

Despite the increased comprehensivity of analysis, not all scanned embryos were fully interpretable. In some specimens the HNF3β marker signal was not strong and was obscured in particular locations meaning not all embryos were scorable for all abnormalities. This is possibly due to incomplete penetration of the antibody or other staining components in some cases. When an embryo was not scorable for a particular abnormality, it was not included in the analysis (Table 2).

It was shown that the dose and timing of exposure of embryos to Adriamycin is critical in producing foregut malformations [45]. The exact preparation of adriamycin, dose and treatment regime used here was that previously shown to have the strongest effect in the Dawrant et al. study (2007c). While the overall effects on the foregut are broadly consistent between studies some differences are likely to be due to differences in the treatment regime. Ionnides at al. reported high incidence (60%) of LTEC at E12.5 with a dose of 4 mg/kg of adriamycin, administered at E7.5 and E8.5 [23]. In contrast, in this work, where a higher dose was given earlier (6 mg/kg at E7 and E8), the incidence of LTEC at E12 was 18% and the incidence of OA was 60%. The very high incidence of OA with upper pouch at E12 in comparison to 15.6% in embryos at E18 seen in an earlier study with the same treatment regime [24] may be explained by the fact that the most severely affected embryos may not survive to E18.

To assess if adriamycin exposure has an effect on the rate of development, embryos were staged according to Theiler morphological criteria and the range of stages at each embryonic day of development were compared between control and adriamycin treated groups. This showed a significant delay in development in treated groups across all days of development with a steady increase in the extent of delay over time. It is recognized that there can be a 6 to 12 hour difference in embryonic development between embryos within the same litter [46] so it is important to compare across a large number of embryos in control and treated groups. The method used here, where a predominant stage was identified for control embryos on each embryonic day and the proportion of embryos at a less advanced stage noted, allowed the increasing delay in treated embryos to be revealed in a robust manner. Because the criteria used to stage the embryos spanned multiple developing systems including facial, brain and limb features, we conclude that this level of adriamycin exposure delays development processes in general. In addition, 3D imaging showed the normal progression of lung development over a series of Theiler developmental stages and a marked specific delay in lung progression in treated embryos relative to overall development judged by other morphological criteria was observed. The observation that in 20% of treated embryos staged at TS20, the trachea and oesophagus had separated whereas at TS18 no separation was observed (an event observed at TS17 in control embryos) indicates that in some treated embryos there may be some recovery in the effects on lung development, however the effects are very severe and the system is unlikely to fully recover.

The notochord is a key embryonic structure common to all members of the phylum Chordata. In higher vertebrates it exists only transiently but has a potent influence as a source of inductive signals patterning adjacent tissues including the neural tube dorsally, the somites laterally and the gut endoderm ventrally [11], [47]–[51]. The cells of the notochord derive from the node during gastrulation and initially form a notochordal plate embedded in the dorsal gut endoderm [10], [11]. The notochord cells reorganize into a rod-shaped structure that detaches from the endoderm, becomes separated from it by intervening mesenchyme, and comes to lie in closer association with the neural tube dorsally [10], [11]. The mechanisms regulating this dorsal delamination of notochord cells in a timely manner are not known. The existence of abnormal ventral ‘branches’ extending from the notochord has previously been noted in adriamycin exposed rat and mouse embryos [25], [43], [52]–[54] indicating that the delamination process does not proceed correctly. Here we observe two clear sets of defects during development of treated embryos (delamination of the notochord and abnormal foregut development) and although these may be caused independently, the close physical association between abnormalities of the notochord and the foregut suggests a causative link whereby abnormal delamination of the notochord leading to ventrally displaced notochord tissue may disturb foregut development, either through mechanical disturbance or by producing aberrant signals misdirecting the patterning of the foregut. In support of this idea there are a number of compelling observations in the literature illustrating notochord defects and problems with development of endodermal tissues; for example the Noggin knock-out mouse, which exhibits features similar to adriamycin treated embryos including abnormal branching of the notochord and also OA/TOF defects of the foregut [11], [27], [31]. These observations suggest that the timing and position of notochord signaling must be closely regulated for correct development of the gut tube.

In terms of molecular players important in signaling from the notochord to the foregut, gene inactivation studies show that Shh is essential for normal patterning of the foregut possibly through Foxf1 activation [30], [40]. We showed that the branched notochord expresses Shh at a high level and therefore branching of the notochord would lead to closer and increased signaling of Shh to the foregut. In the adjacent foregut we observed lack of Sox 2 expression in the dorsal endoderm, abnormal dorsal expression of Shh in the endoderm and absence of the normal ventral bias in the foregut mesenchyme expression of Foxf1 at this level (Figure 8). In the case of noggin knock-out mice, also showing abnormal notochord branches, the Foxf1 expression domain was expanded dorsally around the foregut compared with wild type embryos. Although the patterning defects in these two cases are not identical, both are consistent with the idea that abnormal signaling from ventrally displaced notochord alters patterning of the adjacent foregut. It is not surprising that the defects are different in these cases as although both involve ectopic notochord tissue close to the foregut, in the case of the noggin knock-out this tissue cannot express noggin and the signaling characteristics may therefore differ. In both cases the correct distinction between dorsal and ventral endoderm is lost which may be important for the correct process of separation of dorsal digestive tract and ventral respiratory tract and may lead to the foregut abnormalities of OA/TOF observed in both.

It is intriguing that haploinsufficiency of *Bmp4*, presumably lowering the level of BMP4 produced, can rescue notochord branching and the OA/TOF phenotype in *Nog* null mutants [27], supporting the suggested idea that the precise level of BMP signaling, normally regulated by Noggin, is crucial for correct morphogenesis of the notochord and for foregut patterning. In adriamycin treated embryos BMP4 signalling activity might be disturbed as a consequence of ectopic Shh expression through the notochord branches [30], [55]. This might also lead to reduced expression of Fgfs in the mesoderm [56], [57] supported by our previous observation of delayed mesodermal Fgf10 expression in the adriamycin model [29] and delayed outgrowth of the primary lung buds from the foregut observed here. The disturbed expression of Sox2 in the dorsal foregut endoderm in the region of a notochord branch is interesting in light of recent observations that Sox2 repression in the ventral foregut promotes respiratory identity [58]. It is clear however that a complex balance of inter-regulated signaling pathways is involved in normal development since truncation of the trachea and lung maturation is also affected in mutants with disrupted Wnt signaling pathways [59], [60].

Although the accumulated observations presented here suggest a causative relationship between abnormal notochord branches and foregut abnormalities, this remains an open question. The mechanistic basis of abnormal notochord branching is still not known but the current findings add to the body of data in this area that allow possible mechanisms to be explored [43], [61]. Any model must take account of the various data on implication of molecular players and the association seen here between specific location and heaviness of notochord branches associated with foregut atresia and foregut stenosis. Mechanical disruption of normal foregut development cannot be ruled out and the altered dorso-ventral patterning observed suggests a fundamental signalling defect. This systematic approach, combining a morphological marker and 3D imaging improves our insight into the developmental sequence leading to such foregut abnormalities and affords the opportunity to study them with high precision. The observations confirm previous findings on morphological abnormalities in the adriamycin rat and mouse model and open new question for further directions of molecular study which may bring us closer to understanding the pathogenesis of OA/TOF in humans.

## Supporting Information

Table S1 Scoring of notochord branching and co-incidence of specific foregut abnormalities 2-8 (as per Table 1) in 50 E10 and E11 adriamycin treated embryos.
(DOCX)
