# Supplementary material for: Abnormal Notochord Branching Is Associated with Foregut Malformations in the Adriamycin Treated Mouse Model
Source: PLoS One. 2011 Nov 21;6(11):e27635. doi: 10.1371/journal.pone.0027635 (PMC3221665; doi:10.1371/journal.pone.0027635)
Supplement: Table S1 — Scoring of notochord branching and co-incidence of specific foregut abnormalities 2-8 (as per Table 1) in 50 E10 and E11 adriamycin treated embryos. (DOCX) [file pone.0027635.s001.docx]

Supplementary Data Table 1. Scoring of notochord branching and co-incidence of specific foregut abnormalities 2-8 (as per Table 1) in 50 E10 and E11 adriamycin treated embryos.

| **Position and heaviness of notochord branching** | **Morphological abnormalities (as per table 1)** | | | | | | |
| --- | --- | --- | --- | --- | --- | --- | --- |
|  | 2.Foregut atresia | 3.Foregut stenosis | 4. EA with upper pouch | 5. LTEC | 6. Tracheal atresia | 7.Stomach agenesis | 8.Fistula |
| 1* |  |  |  |  |  |  |  |
| 1* |  |  |  |  |  |  |  |
| 1*****Y |  |  |  |  |  |  |  |
| 1* |  |  |  |  |  |  |  |
| 1* |  |  |  |  |  |  |  |
| 1* |  |  |  |  |  |  |  |
| 1* |  |  |  |  |  |  |  |
| 2* |  |  |  |  |  |  |  |
| 2* |  |  |  |  |  |  |  |
| 2* |  |  |  |  |  |  |  |
| 2* |  |  |  |  |  |  |  |
| 2* |  |  |  |  |  |  |  |
| 2*Y |  |  |  |  |  |  |  |
| 2* |  |  |  |  |  |  |  |
| 2* |  |  |  |  |  |  |  |
| 2** |  |  |  |  |  |  |  |
| 2**Y |  |  |  |  |  |  |  |
| 2** |  |  |  |  |  |  |  |
| 3***Y |  |  |  |  |  |  |  |
| 3**Y |  |  |  |  |  |  |  |
| 3* |  |  |  |  |  |  |  |
| 3** |  |  |  |  |  |  |  |
| 3*** |  |  |  |  |  |  |  |
| 3** |  |  |  |  |  |  |  |
| 3*** |  |  |  |  |  |  |  |
| 3**Y |  |  |  |  |  |  |  |
| 3**Y |  |  |  |  |  |  |  |
| 3* |  |  |  |  |  |  |  |
| 3*** |  |  |  |  |  |  |  |
| 1*,2*,3* |  |  |  |  |  |  |  |
| 1*,2*,3* |  |  |  |  |  |  |  |
| 1*,3** |  |  |  |  |  |  |  |
| 2*,3* |  |  |  |  |  |  |  |
| 1*,2** |  |  |  |  |  |  |  |
| 1*,3* |  |  |  |  |  |  |  |
| 2*,3*Y |  |  |  |  |  |  |  |
| 2*,3* |  |  |  |  |  |  |  |
| 1*,2*,3* |  |  |  |  |  |  |  |
| 2*,3* |  |  |  |  |  |  |  |
| 1*,3* |  |  |  |  |  |  |  |
| 2*,3*Y |  |  |  |  |  |  |  |
| 2**,3* |  |  |  |  |  |  |  |
| 2*,3*Y |  |  |  |  |  |  |  |
| 1*,2*Y |  |  |  |  |  |  |  |
| 1*,2* |  |  |  |  |  |  |  |

For position 1= anterior to the pharynx; 2= level of the trachea; 3= between tracheal separation and stomach. Number of stars represents the relative thickness of the branch. Y= y shaped double branch of notochord
